# Epidemiology of Dementia in Africa

**DOI:** 10.1002/alz70860_105503

**Published:** 2025-12-23

**Authors:** Rufus O. Akinyemi

**Affiliations:** ^1^ College of Medicine, University of Ibadan, Ibadan, Oyo State, Nigeria; Africa Dementia Consortium, Ibadan, Ibadan, Nigeria; Institute of Advanced Medical Research and Training, College of Medicine, University of Ibadan, Ibadan, Oyo State, Nigeria

## Abstract

Approximately 60% of people living with dementia live in low‐ and middle‐income countries (LMICs) and this percentage is expected to increase to 71% by 2050. In Africa, the increase is being driven by population growth, population ageing and escalating burden of cardiovascular disease and other factors. The cost of dementia in sub‐Saharan Africa was estimated at 6.2 billion USD in 2015. Epidemiological studies on dementia are currently growing in Africa. Prevalence rates ranging from 2.3% to 23.0% have been reported, but recent meta – analysis of up to 44 studies yielded a pooled prevalence rate of about 5% and pooled incidence rate of 2% and increasing mortality. Differential prevalence rates have been obtained from multiple studies due to differences in screening tools, diagnostic approaches and criteria and population diversity. Alzheimer's disease, vascular dementia, HIV ‐associated neurocognitive disorder (HAND) and other ADRD subtypes have been reported in Africa, but AD is most common (60%) and vascular dementia constitutes up to 30% of all dementias (compared to 15% among Caucasians). The risk factors for dementia in Africa have been documented from multiple studies and meta‐analyses. Overall hypertension, diabetes, dyslipidemia, low education, social isolation and low socio ‐economic status are prominent risk factors. Using the framework of the Lancet Commission on Dementia risk factors 2024 iteration, preliminary analysis in the ongoing READD – ADSP Study showed that in the bivariate analysis, older age, female sex, hypertension, hearing loss, depression, physical inactivity, low level of education and social isolation were significantly more prevalent among cases compared to the controls while visual loss (self‐reported) was more prevalent in the control population. Factors independently associated with dementia include age (AOR 1.03, *p* <0.05), hypertension (AOR=1.35,) alcohol use (AOR=0.34), depression (AOR=2.60) and visual impairment (AOR=0.62). Comprehensive longitudinal studies with robust and rigorous methodology and equitable regional coverage will provide large datasets on risk factors for dementia in Africa that will enable accurate computation of population attributable fraction (PAF) for the key modifiable risk factors for dementia in Africa. These are critical for developing context – sensitive risk reduction strategies for the African population.

